# Taphonomy and chronosequence of the 709 ka Kalinga site formation (Luzon Island, Philippines)

**DOI:** 10.1038/s41598-020-68066-3

**Published:** 2020-07-06

**Authors:** T. Ingicco, M. C. Reyes, J. de Vos, M. Belarmino, P. C. H. Albers, I. Lipardo, X. Gallet, N. Amano, G. D. van den Bergh, A. D. Cosalan, A. Bautista

**Affiliations:** 1 Département Homme Et Environnement, UMR 7194, Muséum National D’Histoire Naturelle, Sorbonne Université, Musée de L’Homme, 17 Place du Trocadéro, 75016 Paris, France; 2 National Museum of the Philippines, Padre Burgos St., 1000 Manila, The Philippines; 3 Naturalis Biodiversity Center, P.O. Box 9517, 2300 RA Leiden, The Netherlands; 4 Archaeological Studies Program, Albert Hall, University of the Philippines, Diliman, 1101 Quezon City, The Philippines; 5 Max Planck Institute for the Science of Human History, Kahlaische Str. 10, 07745 Jena, Germany; 6 Centre for Archaeological Science, School of Earth, Atmospheric and Life Sciences, University of Wollongong, Wollongong, NSW 2522 Australia

**Keywords:** Archaeology, Natural hazards

## Abstract

The recently described site of Kalinga in the Philippines adds to our understanding of Early-Middle Pleistocene hominin behaviour. Yet, disentangling the natural from the anthropogenic modifications that have taken place in such an old archaeological site is challenging. In this paper we use a set of taphonomic tools at hand to rectify the distortion made by natural processes during the formation of the Kalinga site. From the description of the ribs completeness, surface damages and scattering in the excavation, one can reconstruct the butchery, transport and deposition sequence of the rhino carcass and its post-depositional disturbances and diagenetic evolution of the site. We conclude that the rhino and the stone artefacts potentially used to deflesh the carcass were transported by a mudflow from its butchery place over a few meters only and got stuck and mixed with an older faunal assemblage that was transported by a small stream.

## Introduction

**Figure 1:**
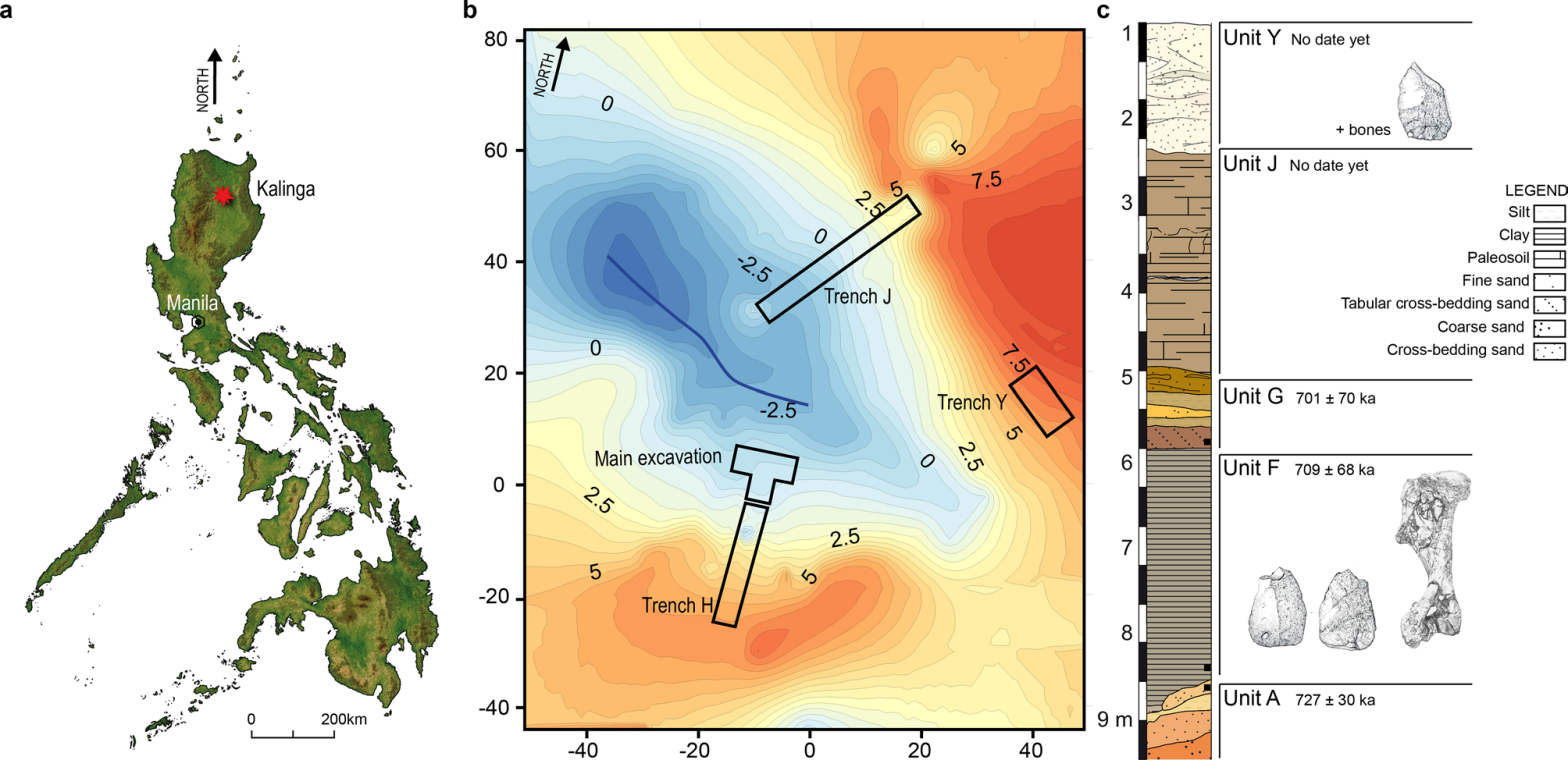
Geographic location of the Kalinga site (**a**), topographic map of the small valley where the excavation took place, pointing at the different trenches and at the seasonal stream (blue line) (**b**) and synthetic stratigraphic profile (**c**) of the site within which two layers delivered archaeological material. The black squares point to dating samples.

Lower and Middle Pleistocene Asian sites opening a window on subsistence behaviour of ancient hominins are scarce. So far, three sites have yielded evidence of clear butchery activities^1^: 800 ka Indonesian site of Ngebung 2^2,3^ and the 412 ± 25 ka site of Hexian^4^ as well as the ca. 300 ka Chinese site of Zhoukoudian locality 4^5^. This scarcity of records of early Palaeolithic subsistence strategies in Asia is made more challenging by the integrity of the archaeological sites as, the older they are, the more likely they are to be heavily disturbed and therefore to be hardly interpreted^6^. The recently described site of Kalinga in the Philippines (Fig. 1a), where the recovery of a 709 ka almost complete disarticulated rhinoceros skeleton showing cutting and percussion marks along with 57 stone artefacts^7^, presents a unique opportunity to study ancient human-animal interactions in this part of the world.

The site of Kalinga is located down North a 7.5 m-high hill, and halfway the southern slope of a small southeast-northwest valley with seasonal run-off waters (Fig. 1b). In the 42 m^2^ excavation named Main Trench on which we focus in this paper, apart from the rhino remains, fossils of tortoise, *Varanus salvator*, *Stegodon luzonensis* and *Cervus* cf. *mariannus* were excavated along with two tektites, several pebbles mostly composed of dacite, and a possible manuport, all of them between 70 cm and 1 m below the present surface within a silty clay layer^7^ (Fig. 1c). This fossiliferous bed referred to as Unit F is uncomfortably overlaying an eroded coarse to medium sandy indurated fluvial layer (Unit A). The archaeological layer of Unit F is in turn sub-horizontally overlaid by a 1–1.15 m thick cross-bedded coarse sandy fluvial layer (Unit G) with silt lenses. Above Unit G is a 2.5 m thick silty pedogenised layer with imprints of rhizomes which are not affecting the underlaying layers (Unit J). The uppermost layer (Unit Y) is composed of cross-beded medium to coarse sands within which a few stone artefacts and poorly preserved bones were recovered. These latter remains which pertain to a different layer are not included in this study. The main bonebed Unit F is briefly interpreted by Ingicco and co-authors^7^ as a mudflow infilling a palaeochannel, most probably following a volcanic eruption subsequent to the butchery of the rhino carcass. More credits are given in this manuscript to support this hypothesis including results of our complete taphonomic analysis.

Fluvial systems have been recognized as a main accumulating agent in archaeological sites^8^. These systems have been studied in detail^9–11^. In the case of Kalinga, a mudflow is suspected to have also played a major role in the accumulation and these systems have been much less studied in archaeology^12^. Geologists and engineers have studied in detail rheological behaviour of mudflows which are usually distinguished by sedimentologists from debris flows by the proportion of coarse elements relative to the clayey fraction^13,14^. These flows have been mainly studied within periglacial environments where solifluxion is common^15^. Yet, mudflows are also common in the tropics following typhoons for instance^13^. While debris flows usually deposit into lobate toes, with forefronts composed of the largest elements within a poorly sorted muddy matrix, and followed by a more fluid flow, in mudflows coarse particles do not accumulate at the flow front^16,17^. Clasts imbrication is common in turbulent viscous mudflows^18^. Such events may result in important modifications of the archaeological material scattering preventing any interpretation regarding hominin behaviour.

In this paper, we aim at disentangling the natural from the anthropogenic modifications that have taken place at the Kalinga site, and as a final goal, to better understand the function of the site and the way of life of the Kalinga hominins. The relational properties of the archaeological materials might be the result of a series of depositional and post-depositional processes making the interpretation of any anthropogenic behaviour difficult^19–23^. In Kalinga, there is so far no evidence that the stone artefacts recovered along with the rhino carcass were the ones used by the hominins to deflesh the bones. Behrensmeyer^9^ identifies two types of sites: allochtonous sites gather assemblages originating from distant places that have undergone significant post-depositional disturbances; and autochtonous sites that are made up of materials recovered where they were deposited or that have undergone only a minimal dispersion at the most, close from their original *locus*^24^. Several tools are at hand to rectify the distortion made by natural processes during the formation of the site^6^. The combination of taphonomy and spatial analysis has been frequently used in the last decade, mostly in the re-evaluation of ancient excavations of open air Lower and Middle Palaeolithic sites from East Africa^20, 24–26^. Such approaches have been much less in use in Asian and Southeast Asian archaeological sites. Bone and stone artefact surface modifications as well as chemical analyses provide some important complimentary information on any biotic or abiotic agents involved in the site formation^2,27^. Here we use this set of tools to make several analytical inferences on mechanical and geobiochemical modifications of the Kalinga site and to propose a hypothesis for its early Middle Pleistocene formation history. Our study focuses on the Main Trench which constitutes the bulk of the archaeological assemblage recently excavated in the Kalinga site. The results provide another look at one of the few and therefore important Early Palaeolithic sites in Asia from which Pleistocene hominin behaviour can be deciphered.

## Materials and methods

Following Schiffer^6^, the disentangling of natural and anthropogenic processes at the origin of the site formation was conducted by looking at different properties of the archaeological objects: size and weight, density, shape, orientation and dip, bone surface damages and accretions.

The fossiliferous Unit F was exposed in the Main Trench and in Trench H (Fig. 1b). This sedimentary unit was excavated with small bamboo sticks made at the site in order to prevent damages on the surface of the bones and stones. A total of 574 bones, 162 stones and 73 lithic artefacts have been recovered so far, all originating from the Main Trench. The three-dimensional coordinates of all artefacts and fossil bones larger than 2 cm were recorded with a Leica© Total station. The orientation, dip and plunge of every bone with length twice greater than their width were further recorded with the same instrument through the measurement of two three-dimensional coordinates at each end of the longitudinal axis of the bones following the recommendations of Dominguez-Rodrigo et al.^24^ and contra Benito-Calvo and de la Torre^26^.

These coordinates were used to produce a two-dimensional kernel density estimation following de la Torre et al.^25^. The optimal bandwidth for the kernel density estimation was calculated from the normal reference distribution by minimizing the mean integrated square error following Scott’s rule-of-thumb approach^28,29^. Density maps were plotted for the whole assemblage and for each type of remains: rhino bones, other faunal elements, pebbles and stone artefacts. Because, in the latter case, the sample size for each of these categories changed with a factor of ten, the optimal bandwidths were independently estimated for each of them. We computed correlations between the density estimates for the four categories of remains in Kalinga. Comparing the densities for each of the categories could nevertheless be limited by the different size sample and the different smoothing used for each of the groups. Comparisons were therefore made on linear models generated for each categories through a pairwise comparison with a 95% confidence level. To avoid any misinterpretation due to sample size, the comparisons were made on the least square means which are means for each category that were adjusted for means of the other categories in the linear model^30^. These results were further presented into a plot as Supplementary Figure 1 comparing confidence intervals for the estimated marginal means. This was achieved thanks to the package Emmeans^31^ in R^32^. With the same goal, a smoothed kernel density was estimated for the categories taken two by two (Supplementary Figure 3).

Because the Kalinga material we are analyzing is composed of very large complete bones of rhino and small (between 20 and 50 mm) artefacts, pattern recognition analysis through quadrats did not appear appropriate as the size of the quadrats could hardly be optimal for all the categories^33^. The search for aggregation and segregation within the Kalinga material was achieved thanks to clustering methods^34^. Partition around medoids (PAM) algorithm was preferred over K-means approach for it is more robust with outliers. Because clustering methods require a-priori knowledge of the number of clusters we are searching for, we estimated the optimal numbers of clusters by comparing 26 indices from eight hierarchical and non-hierarchical clustering algorithms iteratively run with 2 to 15 possible clusters. This was achieved with the NbClust function of the package^35^ of the same name in R. Following de la Torre and Wehr^25^, we also applied several empirical tests of spatial dependence such as nearest neighbor distances (G function), empty space distances (F function) and pairwise distances (K function) from Spastats package^36^ in R as to evaluate the clustering pattern from a theoretical one (Supplementary Figure 2). The G function measures the distribution of distances from an arbitrary event to its nearest event. Ripley’s F function measures the distribution of all distances from an arbitrary point to its nearest event. K function measures the number of events found up to a given distance of any particular event.

The three-dimensional coordinates for the 50 Kalinga remains that were twice longer than wide, were further used to build stereograms with Open-Stereo software^37^ as to visualize the dominant three-dimensional orientation of the archaeological materials (*i.e.* the fabric^38^). Based upon experiments and modelling^39^, a sample of 50 observations is considered as an appropriate sample size. The coordinates were used to compute a singular value decomposition resulting into three normalized eigenvalues (S1, S2 and S3) as to quantify and determine the nature of the fabric (linear, planar or isotropic) following Woodcock^40^ and to visualize it as a two-axis ratio plot in R. From these eigenvalues, a fabric shape parameter (K = ln(S1/S2)/ln(S2/S3)) and a fabric strength parameter (C = ln(S1/S3)) were computed. A K value between 0 and 1, indicates a planar fabric, meaning that the bones are lying on the stratification plane, and above 1, the fabric is linear, meaning that there is a strong main orientation. The greater the C parameter is, the more anisotropic the orientations of the bones are. We applied a series of test for departure from uniformity of cicular data^24,41^. Kuiper’s test is a derivative of the Kolmogorov–Smirnoff test based on the von Mises distribution. The more the angles are equally scattered in all directions (e.g. isotropy), the closer to zero the statistics of the Rayleigh will be. Rao’s spacing test is based on the space between the angles with the consideration that if the angles are uniformly scattered in all directions, then the arc lengths between any two of the angles should have a particular type of distribution. A large Watson test’s statistics is expected from non-uniform directions. These tests were achieved with the package Circular^42^ in R.

Because the pebbles recovered at the site have predominantly globular shapes (with near equal a, b and c axes), a fabric could not be computed. A total of 93 pebbles mostly dacites were weighed using a digital scale. The scattering of these pebbles per weight was used to build bubble graph to visualize their distribution per weight and types. Similarly, no fabric could be computed on the stone artefacts. Because all the stone artefacts look very fresh and no refittings were found, no taphonomic analysis such as the one developed by de la Torre and co-authors^25^ was performed on them. We computed atheoretical regressions on each dataset through a locally weighted scatterplot smoother (LOESS) fitting method, over second order polynomials. LOESS are non-parametric regressions combining several regression models based on the *k* nearest neighbour method. The advantage of LOESS is the weight it gives to local samples which are better representations of the complexity of a dataset, something that a simple linear regression would not be able to meet.

The fabric results, the density kernels and the clustering results were all compared with the topography of the excavated surface on which the archaeological remains were laying. A Digital Elevation Model (DEM) has been produced by interpolation through a kriging method from 67 three-dimensional coordinates recorded in the Main Trench on the erosional surface that cuts down into Unit A. This surface of contact corresponds to a main erosional channel cutting down into sandy Unit A. After filling the potential remaining holes in the DEM with another interpolation thanks to the “Fill sinks” function of SAGA within the freeware QGIS 3.3.4^43^, a hydrological model was produced from this raster with the functions r.flow of GRASS from the same freeware, which evidenced the run-off waters based on the slope of the terrain; Strahler stream order resulting in hierarchized flow accumulation areas was computed with the function “Strahler order” of SAGA within the same freeware. From these latter, catchment areas were estimated for the contact surface between Unit A and F. Hydrological models have proven to be helpful in the understanding of complex fluvial processes^22,25,44–46^. Slope and aspect maps, as well as vector plots have further been produced from the DEM in R and the use of the package rasterVis^47^. Slope can be defined as the plane at a tangent to a point on the surface, while aspect is the direction of the plane with respect to the North^48^. The directions of the slope and related runoff waters were visually compared with the fabric results. The density and clustering patterns were confronted to the hydrological model. Four linear regressions were computed. One (Eq. 1) to obtain the dominant orientation of the elongated bones of the rhino, another (Eq. 2) to get the main direction of the main flow accumulation area, a third one (Eq. 3) was computed for the southernmost portion of the main flow accumulation after observing that this flow is changing direction at the excavation around coordinates easting = − 6.5, northing = 1.5. Finally, a linear regression (Eq. 4) was computed for the northernmost portion of the main flow accumulation area, after the curve. The coefficients of each of these three linear regressions obtained for the main flow accumulation were compared with the coefficients of the linear regression of the elongated bones through a Pairwise comparison with a 95% confidence level. The clustering was also visually evaluated at the light of the estimated catchment areas.

Every single finding larger than 5 cm was drawn on graphing paper at a scale of 1:10, and several photographs of the excavation were taken at the end of every working day.

The rhino bones were retrieved one by one after having been first protected with a layer of petroleum jelly, a layer of plastic bags and an outer layer of plaster of Paris supported by bamboo sticks to consolidate the samples for transport. Fine cleaning with water and a soft brush and consolidation of the bones with a 20% paraloid B72 thermoplastic resin solution were conducted in the zooarchaeology laboratory of the Archaeological Studies Program of the University of the Philippines Diliman using a magnifying glass.

**Figure 2:**
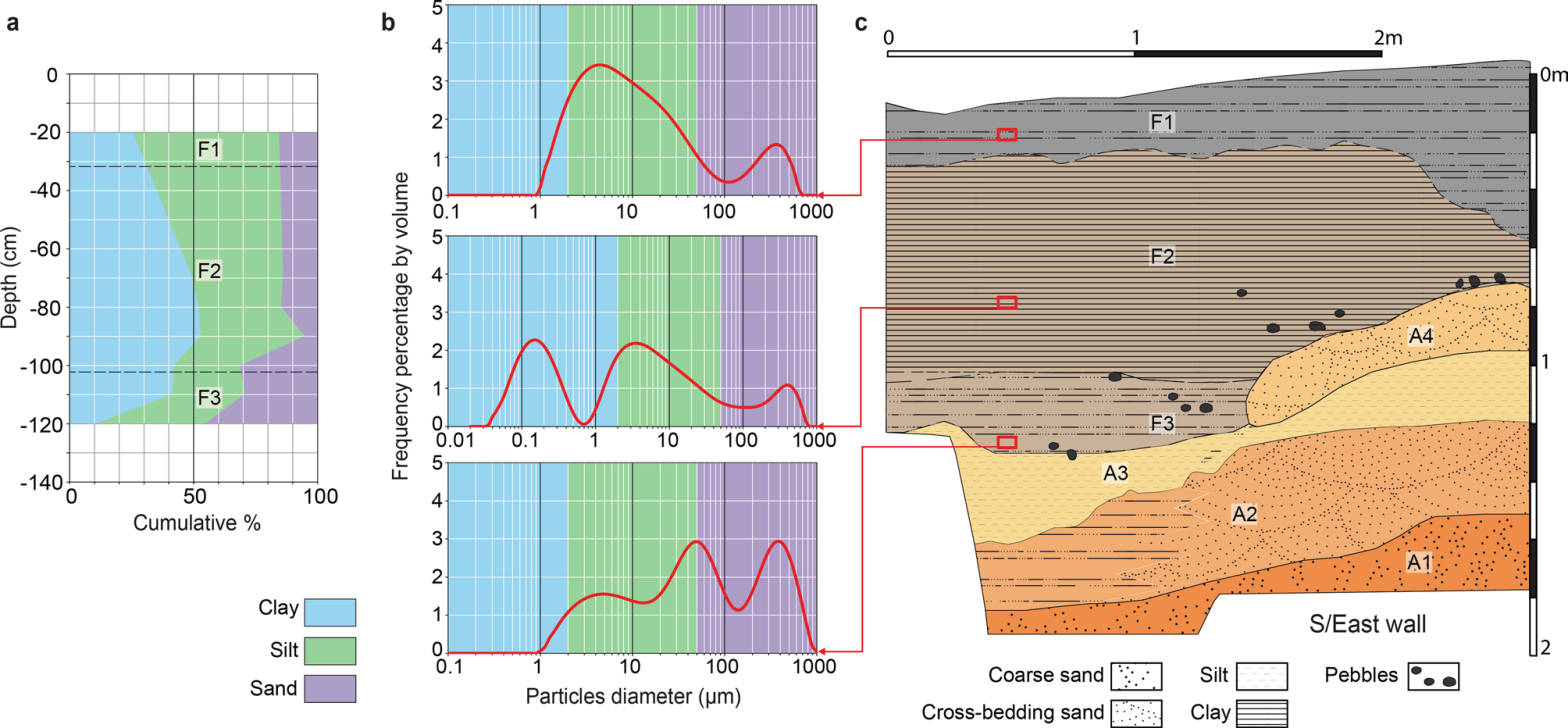
Grain-size analysis of the fossiliferous Unit F, with cumulative grain-size distribution (**a**) through depth (**a**) based on nine sedimentary samples of which three are detailed (**b**), and subdivision of the Unit F based on the grain-size from the Main excavation South/East stratigraphic profile (**c**).

Nine samples of sediments were taken from bottom to top of the fossiliferous Unit F (Fig. 2a–c). Grain size analyses were performed at the sedimentology laboratory of the School of Earth and Environmental Sciences at the University of Wollongong, Australia. Small amounts of each sample were placed in plastic flasks with distilled water, which were then placed in an ultrasonic bath to loosen the sediment over a period of 2 h. The samples were then sieved through 1 mm mesh and analysed with a Malvern Mastersizer 2000©.

Quartz crystals are known in two polymorphs, the low temperature formed alpha-quartz and the high-temperature formed beta-quartz. Bipyramidal quartz shapes are alpha-quartz paramorphs of the cooled down hydrothermal beta-quartz. The search for bipyramidal quartz and their sorting from other polymorphs of quartz therefore provides an indication on the environments at the origin of the mineral composition of the site’s sediments. Furthermore, due to their commonality and hardness of 7 on Mohs scale, detrital quartz grain surfaces further record through the type and degree of erosion, the different environments they encountered before accumulating in the archaeological site^49^ (e.g. wind versus water-transport).

A sample of 100 g of sediments was collected from the fossiliferous layer F2. The sediments were first wet-sieved with a sieving column made of six different mesh sizes: 0.05–0.315 mm, 0.315–0.5 mm, 0.5–1 mm, 1–1.25 mm, 1.25–1.5 mm, 1.5–2 mm^50^. Each granulometric fractions was dried in an oven at 40 °C. The light fraction (quartz and feldspar) was separated from the heavy fraction (zircon, olivine, green hornblend, etc.) using bromoform, a 2.89 density liquid on the 0.315–0.500 mm dry refusal. Ultrasonic cleaning was used to prevent any contamination. The 0.315–0.500 mm light fraction was sorted out under a binocular microscope by types (feldspars, quartz, and other minerals). A total of 7,168 minerals were identified among which 14% (*N* = 1,004) were positively identified as quartz. Finally, the quartz were regrouped into five classes based on their morphology, e.g. shape and degree of erosion:

- Fresh bipyramidal quartz, with fresh pyramid shape appearance, well-developed quartz crystal and the absence of any chemical dissolution;
- Semi-angular to rounded bipyramidal quartz, characterized by smoothed angles and edges;
- Well-rounded bipyramidal quartz, with rounded edges and polished surface indicative of a long fluvial transportation^49^.
- Angular quartz, which are broken bipyramidal quartzes with only a limited degree of abrasion.
- Sub-angular to rounded quartz, which are broken angular quartz with rounded edges, usually crescent- shaped, and are the result of an advanced stage of grain erosion. Some of them preserve a remaining bipyramidal shape.

While broken quartz are the result of fluvial transport in addition to at least initial aeolian transport, unbroken quartz might result from aeolian transportation without any fluvial action^49^. A total of 40 quartz crystals were selected within the five classes for additional exoscopic observations under a Hitochi TM3000 scanning electronic microscope (SEM) at *Musée du Quai Branly-Jacques Chirac* in Paris to better identify the nature of their mechanical wear.

Elemental analyses of Unit F sediments were carried out using a XGLAB© Elio portable X-Ray fluorescence (XRF) spectrometer at the *Plateau Analytique du Muséum au Musée de l’Homme*. The system is composed of a X-ray source based on a Rh anode and a Silicon Drift Detector with an active area of 25 mm^2^, the source emission is collimated creating an analysis spot diameter of 1.2 mm on the sample at a working distance of 1.4 cm. Analyses were performed at 20 kV and 200µA, with an accumulation time of 300 s. The chemical composition of the two samples from the Main Trench were analyzed independently, one on raw bloc of sediments and the other on a sampled part of this bloc, powdered and homogenized in an agate mortar for 10 min then pressed into a pellet inside a mold under 20 ton by cm^2^.

## Results

The fossiliferous Unit F is approximately 2.5 m thick, but the upper part of the unit is partly removed by recent erosion and partly overprinted by soil (called Sub-unit F1). Unit F is predominantly made up of silty clay, with variable admixtures of sand and pebbles (Fig. 2b). These pebbles are always matrix supported and no pebble lag deposit is developed. However, pebbles tend to be concentrated in the lower 10–40 cm of Unit F, which appears to be slightly sandier as well. This coarser-grained basal interval (referred to as Sub-unit F3) could locally be distinguished macroscopically but is also evident from grain-size analyses. The concentration of sandy particles in F3 might be related to the uptake of clasts from the underlaying sandy fluvial deposit Unit A. The cumulative proportion of grain size along the stratigraphic profile (Fig. 2a) shows the gradual transition from sand in F3, through silty-clay in the middle portion (referred to as Sub-unit F2), to clayey-silt in the upper part (referred to as Sub-unit F1).

**Figure 3:**
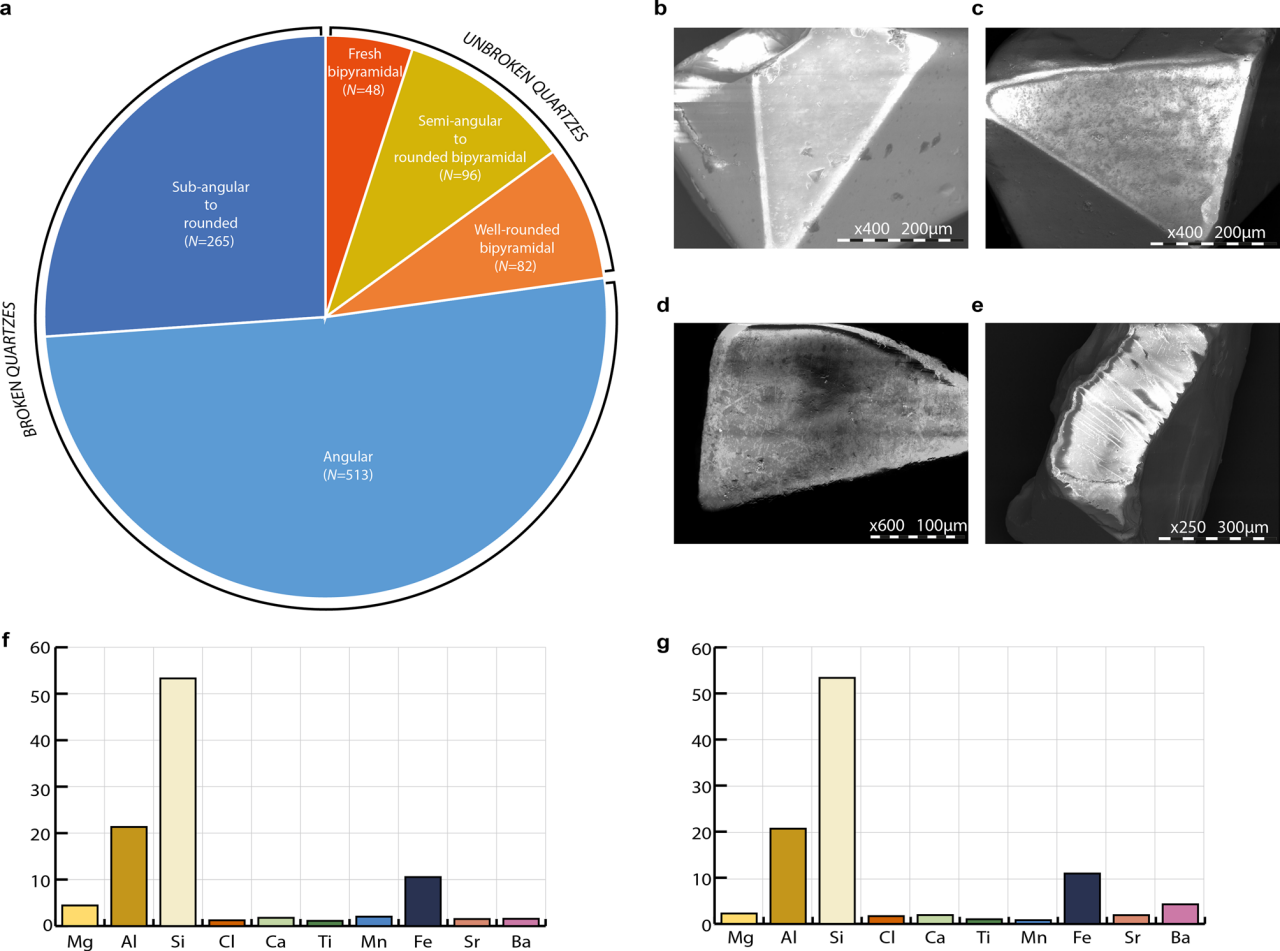
Pie chart of the quartz erosional states (**a**), with details on aeolian marks (**b**), aeolian cupules (**c**), fluvial erosion on an angular quartz (**d**), fluvial erosion on a semi-angular quartz (**e**), and XRF on block (**f**) and pastille (**g**) atomic composition of the Unit F sediments.

Figure 3a summarizes the proportion of each categories of quartz shapes. Unbroken bipyramidal quartz (angular + rounded) account for 23% of the assemblage while broken bipyramidal quartz, resulting from a more complex history, account for 77% of the whole sample. The majority of fresh and semi-angular to rounded bipyramidal quartz observed under the SEM show a few but clear cupules corresponding to aeolian mechanical marks (Fig. 3b,c), due to contact and friction during wind transport. These grains show no additional marks on their surface confirming the absence of any fluvial agent in their transportation and therefore their simple history. The angular and sub-angular to rounded grains likewise do not present a lot of marks. There are some rare examples of parallel striations (Fig. 3d,e) as a consequence of water transportation. A small proportion of these broken grains has, at least, been fluvially transported after the first deposition of strictly aeolian volcanic quartz.

**Figure 4:**
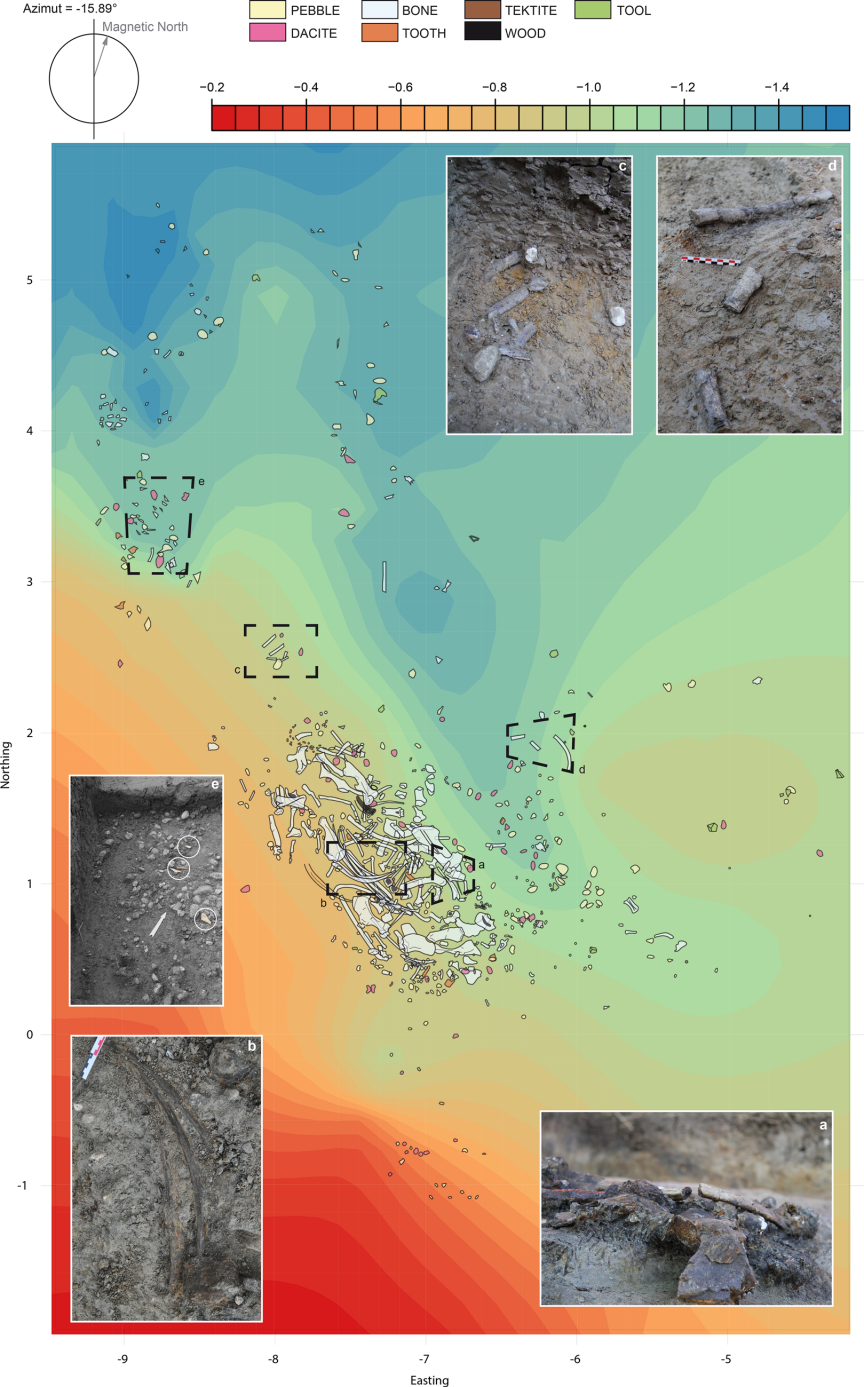
Two-dimensional distribution of the Main Trench excavation findings overlaying the topographic map of the contact surface between the silty clay fossiliferous Unit F and the underlying sandy Unit A, with photographic details of some of the ribs as found in the excavation; A rib transversally broken under sediment pressure (**a**); three almost complete ribs still in anatomical connection (**b**); two ribs that broke in the sediments and for which each resulting fragments slightly shifted one from the other (**c**, **d**); small triangular rib fragments (circled) with fresh edges; scattering of the archaeological findings from Unit F (**e**).

**Figure 5:**
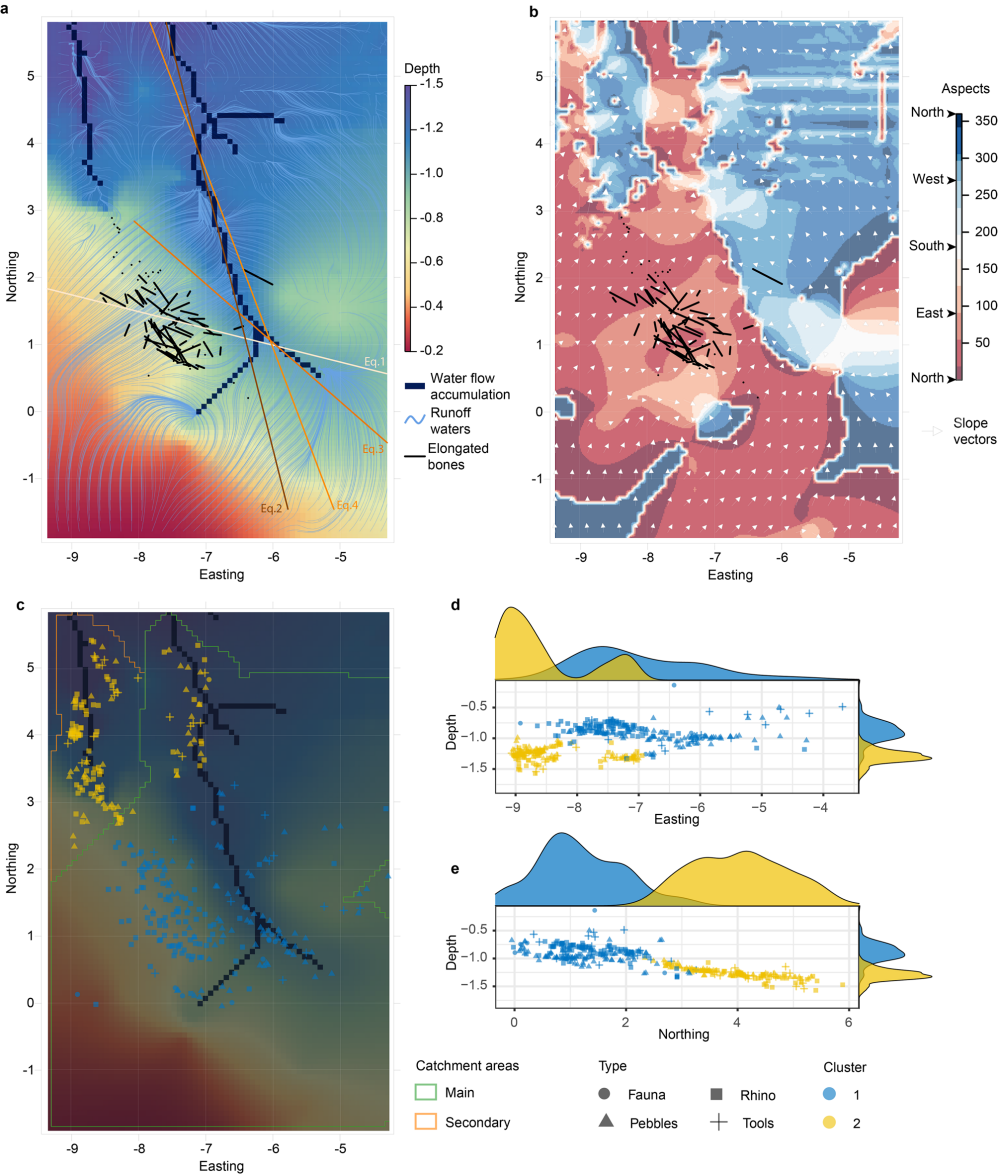
Scattering of the Kalinga assemblage. Topographic map pointing to the main and secondary palaeochannels, runoff waters and elongate bones (**a**). Aspect map and vector slopes of the topography (**b**). Clustering of the assemblage over the topography and catchment areas calculated from the flow accumulation areas (**c**). Vertical dispersion of the cluster along the easting (**d**) and northing (**e**).

The topography of the contact surface between the lower sands (Unit A) and the fossiliferous clay (Unit F) highlights the presence of a main palaeochannel oriented Southeast–Northwest in the southeastern part of the excavation and South–North in the northern part of the excavation area (Figs. 4, 5a,b). Although one cannot measure the width of the main palaeochannel bed, it is clearly corresponding to a small stream as it is further evidenced by the small catchment area. A secondary and smaller palaeochannel, independent from the main one, has also been evidenced in the north-westernmost part of the excavated area (Figs. 4, 5a,b).

**Figure 6:**
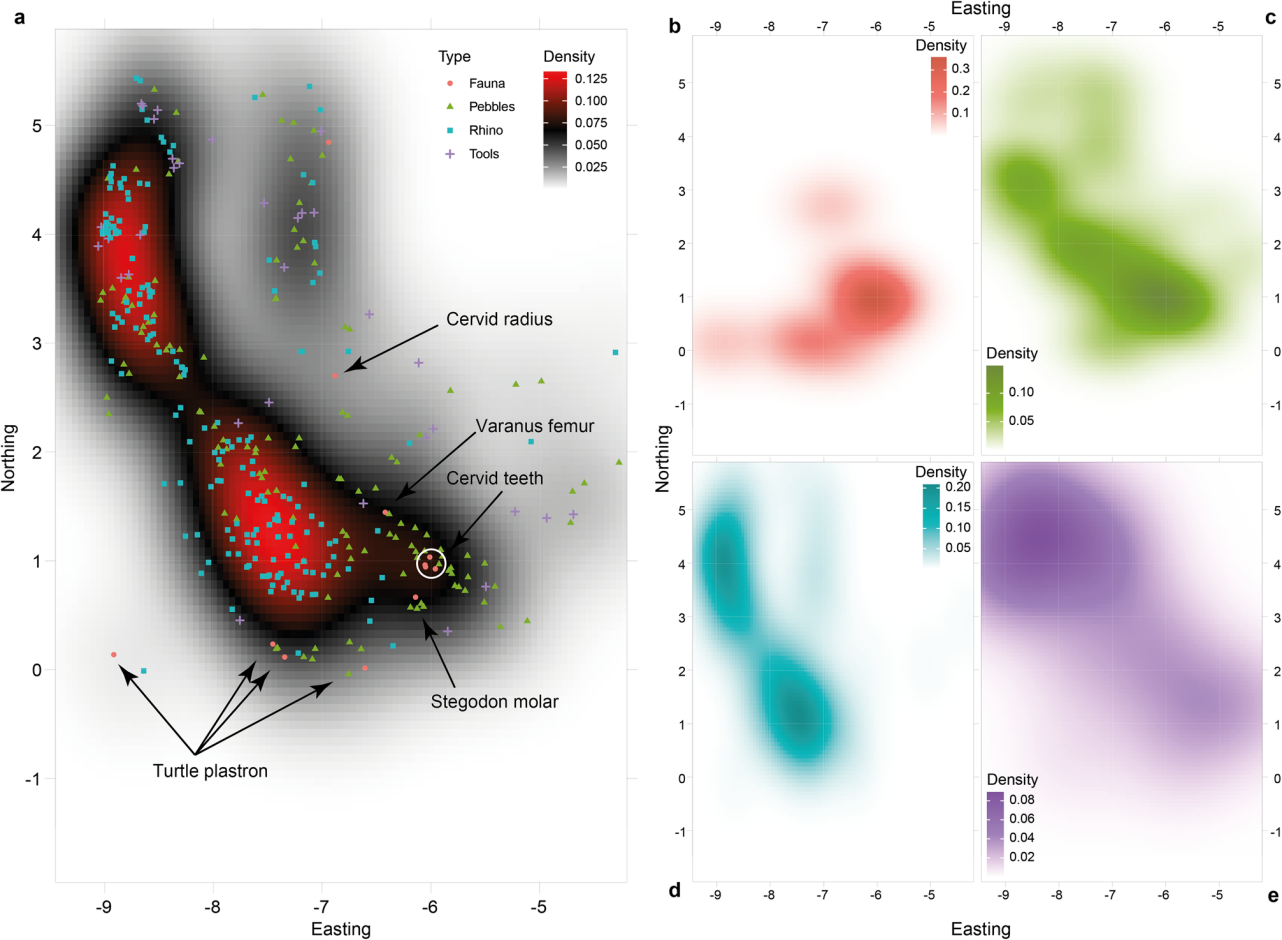
Kernel density estimates for the whole assemblage (**a**), and detailed for faunal remains (**b**), the pebbles (**c**), the rhino bones (**d**) and the stone artefacts (**e**).

The density plots (Fig. 6a–e) show that the Kalinga remains are concentrated in two main areas, one close to the change of direction of the main palaeochannel, the other aligned with the secondary palaeochannel. In details, this configuration is observed for each of the categories taken one by one, the rhino bones, the pebbles and the stone artefacts, at the exception of the other faunal remains which were recovered directly from the bed of the main palaeochannel for most of them (Fig. 6a). The disposition of the two main density areas for the rhino remains, the pebbles or the stone artefacts spreads across the two catchment areas. A third, less dense area within which a few rhino bones, stone artefacts, pebbles and one non-rhino bone were found, is located in the northernmost part of the main palaeochannel (Fig. 6a). The correlations between each categories’ density are summarized in Table 1. The rhino density is hardly correlated with the other faunal remains density (r2 = 0.18). It is slightly better correlated with the stone artefacts (r2 = 0.39) but is at best with pebbles density (r2 = 0.64). Yet, this appears as an apparent contradiction as pebbles are also best correlated with the other faunal remains (r2 = 0.67). The large spreading of the pebbles in any point of the excavation may explain their good correlation with rhino and other faunal remains although those two latter are hardly correlated with each other. This large spreading is clear when one contrasts the density distributions of each category taken two by two through a smoothed kernel density estimates (Supplementary Figure 3). The stone artefacts are slightly negatively correlated with the other faunal remains (r2 = -0.07) and slightly positively correlated with the pebbles (r2 = 0.26). Although the stone artefacts are better correlated with the rhino bones, the value remains relatively low. Yet, comparison of the two categories through estimated marginal means and a pairwise comparison show that the distinction between the rhino bones and the stone artefacts is not significant (Table 2 and Supplementary Figure 1). Therefore, the two categories are partly aggregated, but the artefacts are more densely within the north-eastern kernel unlike rhino bones which are more densely concentrated in the main kernel of the excavation. The contrast between the density distributions shows that the smoothed kernel density between the stone artefacts and the rhino bones is the most linear one among all the compared categories at the exception of the pebbles contrasted with the other faunal remains (Supplementary Figure 3).

**Table 1:** Correlation coefficient for the density distributions.

	Fauna	Pebbles	Rhino	Tools
Fauna	1.00000000			
Pebbles	0.67410293	1.0000000		
Rhino	0.17613652	0.6403777	1.0000000	
Tools	− 0.07252056	0.2600912	0.3886822	1.00000000

**Table 2:** Pairwise comparison on the densities per category. Lower triangle are p-values (delta = 0.05) and upper triangle are estimates.

	Fauna	Pebbles	Rhino	Tools
Fauna		− 0.8766	− 1.2447	− 0.9211
Pebbles	0.9763		− 0.3680	− 0.0444
Rhino	0.9977	0.9882		0.3236
Tools	0.9790	0.4863	0.0145	

Several tests of spatial dependence were applied to examine whether or not the distribution of the archaeological material in Kalinga is random or clustered. Each of our empirical functions (Supplementary Figure 3) is greater (G and K function) or smaller (F function) than the theoretical one which points to the existence of a cluster pattern as the nearest neighbour distances in the point pattern are shorter than for a Poisson process. According to the majority rule, after comparing 26 indices from eight hierarchical and non-hierarchical clustering methods, the best number of clusters for the whole assemblage was estimated to 2 (Fig. 5c–e). About half (53%) of the rhino bones are part of cluster 1 while the other half (47%) are part of cluster 2 (Table 3). 43% of the stone artefacts belong to cluster 1 and 57% to cluster 2. As of the pebbles, two-thirds (67%) are part of cluster 1 and the other third (33%) is part of cluster 2. All the isolated faunal remains others than the rhino are part of cluster 1 at the exception of one fragment of a long bone. A closer look at the distribution patterns (Fig. 5c) shows that cluster 2 is divided into two smaller populations corresponding to the two distinct palaeochannels, one along the flow accumulation line of the secondary catchment area and the other along the northern end of the accumulation flow line of the main catchment area. We tried to repeat the analysis with three clusters to test whether those too smaller groups within cluster 2 would be segregated. Yet cluster 2 remained unchanged with three clusters. Cluster 3 was a subdivision of cluster 1 only. The clustering analysis do not recall the catchment areas of the excavated surface. In cluster 1, some of the artefacts also follow the stream bed of the main catchment area in its northern portion. They are mostly pebbles and isolated faunal remains for only two stone artefacts and one rhino bone. All the best preserved rhino bones among which are the elongated ones we used in the fabric analysis are part of cluster 1. Cluster 2 concentrates the smallest rib fragments which accumulated in the lowermost depressed area of the topography on the north-western part of the excavated surface.

**Table 3:** Percentages of classifications per clusters for each of the categories.

	Cluster 1	Cluster 2
Fauna	92	8
Pebbles	67	33
Rhino	53	47
Tools	43	57

**Figure 7:**
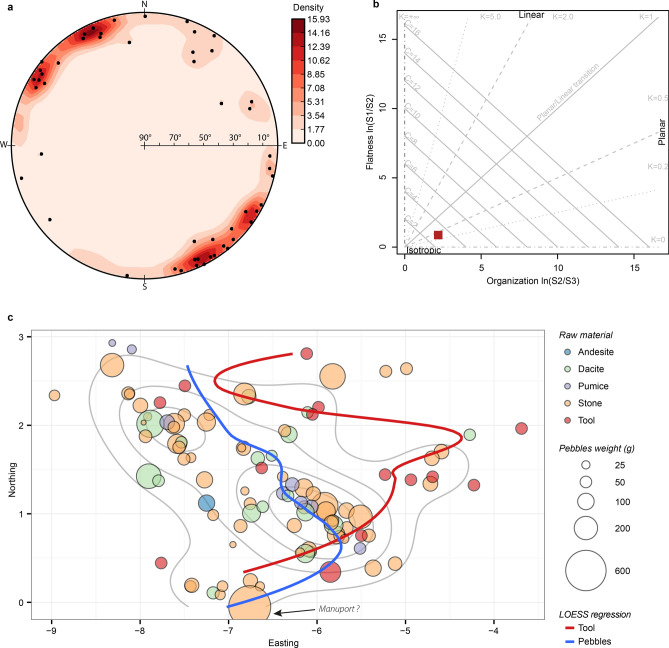
Orientation pattern of the excavated material from Unit F. Stereonet based on 51 elongated rhino bones (**a**); two-axis logarithmic Woodcock diagram (**b**); Bubble plot and LOESS regression curves of the pebbles and stone artefacts distributions by weight and type overlaying kernels of density calculated on the pebbles (**c**).

Most of the best preserved rhino bones were found stuck onto the western bank of the main palaeochannel (Fig. 5a, b) and all have a main Southeast-Northwest orientation (maximum densities at 154° and 334°, mean direction at 254°, radius of confidence at 5% of 41°). The Woodcock diagram (Fig. 7b) and the related strength (C = 3.06) and shape (K = 0.40) parameters underline the isotropic but planar fabric of the rhino bones at Kalinga site. The orientation of the bones has a diametrically bimodal pattern. Therefore, the mean angle is orthogonal to the dominant orientation which problematic for the calculation of the vector of magnitude^39^ and for the statistical tests (Supplementary Table 1) based on the mean, especially for the Rayleigh test which is searching for rejection of the unimodality (e.g. isotropy)^51^. Hence, it is not surprising, with bimodal data, that this is the only test where the main orientation is rejected (Table 4)^45^. Rejection of the null hypothesis by Rayleigh tests for planar fabrics such as ours has already been observed^52^. All the other tests, with low p-values, confirm the main orientation and reject the isotropy (Table 4). This main orientation is also evident on the stereonet (Fig. 5a) which further shows that the rhino bones were horizontally deposited as all the dots are scattered on the periphery of the diagram which indicates a dip close to zero. The main orientation is perpendicular to sub-perpendicular to the slope and therefore to runoff waters (Fig. 5a, b). The main orientation of the rhino elongated bones further significantly differs from most of the main palaeochannel course (Eq. 2 and 4; Table 5), but for its southernmost portion (Eq. 3). Although the two linear regressions (Eq. 1 and Eq. 3) have an angle of 27° between, their equations are not statistically different (Table 5).

**Table 4:** Statistical tests on circular data from the Fabric analysis.

Test	Statistic	P-value
Kuiper's test of uniformity	2.0809	< 0.01
Rayleigh test of uniformity	0.1443	0.353
Rao's spacing test of uniformity	161.3779	< 0.01
Watson's test for circular uniformity	0.1821	0.05 < P < 0.10

**Table 5:** Linear models for the elongated rhino bones and for the main palaeochannel and their relational properties.

Reference	Description	Regression	Angle with Eq. 1 (in degrees)	Pairwise comp. with Eq. 1		
				Standard error	t-ratio	p value
Equation 1	Linear model for the elongated bones	y = − 0.24 * x – 0.46				
Equation 2	Linear model for main flow accumulation	y = − 0.24 * x –14.43	55	0.148	16.255	< 0.0001
Equation 3	Linear model for the southernmost part of the main flow accumulation, for any waypoints greater than x = − 6.5	y = − 0.63 * x –3.75	27	0.389	1.620	0.3694
Equation 4	Linear model for the northernmost part of the main flow accumulation, for any waypoints smaller than x = – 6.5	y = − 3.75 * x –24.03	62	0.270	13.899	< 0.0001

Figure 7c schematizes the disposition of the stone artefacts among the other pebbles sorted by type of material and weight where the best preserved and presumably less transported rhino bones were recovered. The kernels of density of these pebbles are also figured on this diagram. While the dispersion of the pebbles, whatever their type and weight is, recalled the main Southeast-Northwest orientation of the rhino elongated bones, the stone artefacts do not seem to follow this major axis. They were mostly scattered at the periphery of the pebble and rhino kernels of density, although a few stone tools were situated in the central kernel area. Also positioned at the periphery of the kernels is the 650 g pebble we interpreted as a manuport because no other stone in the assemblage exceeds 200 g. The LOESS regression curve (Fig. 7c) for the stone artefacts differs from the one for the pebbles confirming the different patterning between the artefacts and non-cultural stones. The pebbles and the stone artefacts dispersion therefore most likely resulted from a different history, which was also suspected from the comparison of the density patterns for those two categories.

Beyond this general overview of the archaeological material dispersion, some detailed, local aspects of the excavated area are also informative. The rhino ribs were numerous at the site and one can expect, from their general similar morphology that they would result from the same patterning of transport and deposition^53^. Yet, several differences were noticeable. While some of the ribs were complete or nearly complete, some others were highly fragmented. Some of the complete ribs were still clustered together (Fig. 4b) meaning they were still attached during their transport and were therefore not modified by hominins. Some of the larger fragments of ribs were broken transversally to the bone major axis and the fragments refitting each other were almost in anatomical connection, meaning that these ribs broke in the clay, under the pressure of the sediments^54^, after their transport and deposition (Fig. 4a). Additionally, in two cases, some other large fragments of ribs also broke within the clay after they were deposited, and each fragment shifted up to 70° from its anatomical connection (Fig. 4c, d). Finally, four of the smallest rib fragments differed from others by their non-transverse break. One can therefore suspect they did not break like any other ribs within the clay. These *ca*. 5 cm fragments were triangular in shape with strait and sharp edges and devoid of any scratches on their surface, like fresh green ribs broken by fallen large blocks^54^^p.294^. Yet, because large blocks are absent in Kalinga site and vicinity, these fragments are probably not the result of any natural fractures. Furthermore, because large carnivores are absent, not only in Kalinga but in the whole Philippines, one can suspect that these small triangular fragments were likely of anthropogenic driven percussions^54^^p.295^. Interestingly, those triangular rib fragments were all located in the north-western corner of the excavated area, in cluster 1, the farthest away from the main palaeochannel bed, and beyond the palaeochannel bank where most of the rhino carcass larger and better preserved bones lay (Fig. 4e).

**Figure 8:**
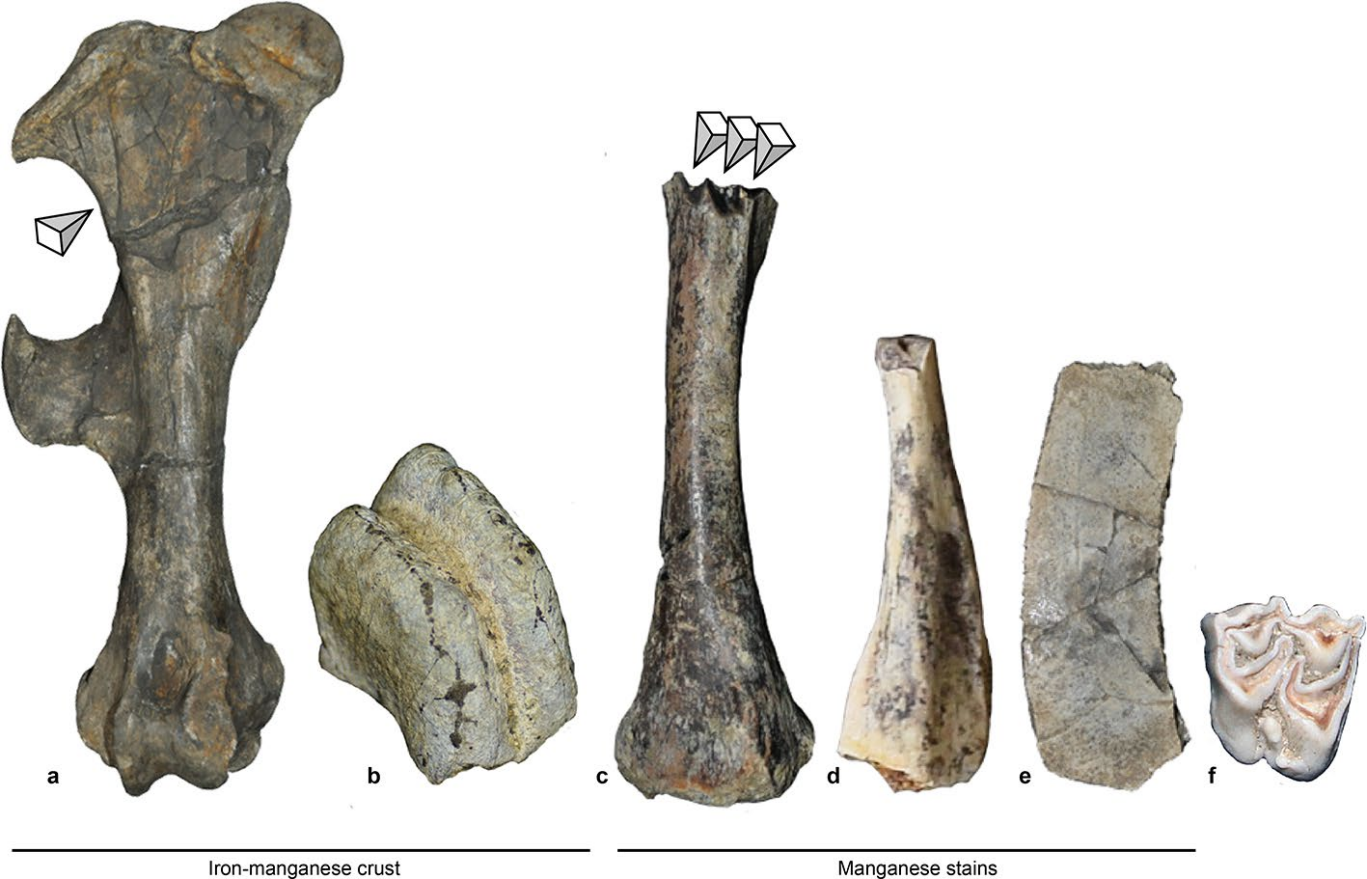
Bone surface characteristics from the Kalinga assemblage. Dark brown and orange rhino femur with black crust and crushed shaft under pressure (arrow) (**a**); Stegodon molar fragment with black crust (**b**); *Varanus salvator* femur with rodent gnawing marks (arrows) (**c**); *Varanus salvator* fibula with manganese stains (**d**) slightly more developed than on the turtle plastron (**e**), while they are absent on the cervid molar (**f**).

The mechanisms affecting the transport of the rhino skeleton are also clear from the bone surface modifications. Ingicco and co-authors^7^ mentioned four ribs marked by thin, multiple and multidirectional scratches interpreted as either abrasion by sediment or trampling or the combination of the two. The abrasion marks were located on the ventral surface of two ribs, on the lateral surface of one rib and on the anterior surface of another rib, and, as such, differs from the anthropogenic cut marks. The two rhino femurs further shown clear compression marks on their shaft upper half (Fig. 8a).

Another unreported yet non-anthropogenic taphonomic agent was recorded through rodents gnawing marks on the upper extremity of a *Varanus salvator* femur shaft (Fig. 8c), which is an indirect evidence for the early presence of micromammals in the Philippines, and that this monitor lizard bone had lain on the ground for a while before being buried by the silty-clay. Furthermore, the *Varanus salvator* fibula is the only bone remain in the whole assemblage of Kalinga showing a shiny polished and slightly smoothed surface we relate to water abrasion. The general colouration of the isolated faunal remains ranges from whitish to light grey and differ from the dark brown and orange colouration of the rhino fossils at the exception of the brown colouration of the *Varanus salvator* femur. The *Varanus salvator* and turtle remains further had some manganese stains tinting their surface at different stages of development (Fig. 8c–f), but nothing like a crust.

Several rhino bones were coated by a hard black crust which was sometimes one centimetre thick. This black crust, when crushed, had some dark brownish tints. Ingicco and co-authors^7^ already noted the presence of black stains inside some of the cut marks left on the rhino bones, although the black crust and black stains may not be related. No black crusts were observed on any of the other faunal remains at the exception of a stegodon molar fragment (Fig. 8). Yet, this fossil had a general whitish colouration unlike the rhino teeth. Like the black crust, black minerals are observable with a naked eye within the silty-clayey matrix of the fossiliferous Unit F. The elemental composition of this clay, obtained by XRF, leaves little doubt that the black minerals in the sediments and black crust on the rhino bones are a mix of iron (Fe) and manganese (Mn) (Fig. 3f, g). Indeed, although Mn is recorded, it only accounts for 1% to 5% of the major elements’ quantification while Fe accounts for 10% to 17% of the atomic composition of the sediment, either measured on block sample or pellet. Although no micromorphological analysis was undertaken, the black colour with brownish tints of the crust is characteristic of reduced ferrous iron (Fe^2+^) mixed with manganese oxide into ferro-manganese concretions, which form under anoxic environments (presence of water and absence of O_2_) and low pH conditions^55,56^ such as in fluvisols and redoxisols^57,58^. The amount and hardness of the hydromorphic crust is directly related to the presence of a significant amount of water in the clay over a prolonged period^58^. Fe is the third most common oxide in Unit F after Silicon (Si) and Aluminium (Al). The high proportion of Si in the clay is most certainly due to the presence of quartz in the matrix.

## Discussion and concluding remarks

From the description of the ribs completeness, surface damages, scattering, orientation and clustering in the excavation, one can reconstruct the butchery, transport and deposition sequence of the rhino carcass and its post-depositional disturbances and diagenetic evolution of the Kalinga site. Additional analyses of the archaeological material distribution and nature of the sediments also contribute to the understanding of the taphonomic processes involved in the formation of the Kalinga site.

In nature, the fabric of a mudflow deposit is the result of the original viscosity and velocity of the mudflow, as well as the moment when the flow stops. Each of these parameters is difficult to precisely identify at the archaeological level. Yet, Lindsay^59^ notes that the gentler the slope is, the more laminar and the shorter the distance of the mudflow will be. Hence, the apparent horizontal contact between the bonebed and the underlying sandy Unit A, the completeness of the rhino carcass, the small 9 m^2^ area where the bones were retrieved, the size diversity of the clasts with very small fragments to very large complete bones of the rhino and the limited natural modifications of the bones all support the idea that the flow was laminar, of high viscosity and low velocity, and that its origin was parallel to the mode of the fabric which itself partly followed the main palaeochannel present there, and that it quickly came to a halt at the site.

### Death and butchery of the rhino

There are at present no clues on what caused the death of the rhino. The attrition pattern of the molars described by Ingicco and co-authors^7^ shows the individual was at an adult stage 6 following Hillman-Smith et al.’s classification^60^ but this is no evidence for its natural death due to advanced age nor for its accidental death due to hominins. No large carnivores have been found at the Kalinga site so far, which is expected in such oceanic islands where faunas are usually unbalanced^61^. In good agreement with this observation, butchery marks were observed at the surface of the rhino bones, suggesting that hominins had a primary access to the carcass.

The butchery is clear as its processing sequence can be confidently estimated. Only a part of the carcass was butchered. Some of the ribs moved together as they were in anatomical connection at the excavation area, which means they were still attached by soft, non-butchered tissues. These remaining soft tissues might have generated anoxic conditions after the deposit and burial. The presence of anthropogenic cut marks described in details by Ingicco and co-authors^7^ on other ribs is evidence that some of the flesh was removed on these specific bones by hominins. Furthermore, four small triangular rib fragments concentrated on the northwestern corner of the excavation, most likely resulted from anthropogenic percussion prior to their transport. Similarly, the percussion marks on the two rhino humeri described previously^7^ are evidence that these bones, like some of the ribs, were defleshed and then tentatively broken by humans most likely to retrieve the marrow. The presence of abrasion marks on the surface of four ribs is further evidence they were defleshed before their natural transport. The fluvial quartz from the sandy Unit A underlying the Unit F at the basis of which the rhino bones were found, are the most probable cause for the abrasion marks at the surface of the ribs.

### Transport and deposition

Following the butchery, the rhino carcass was transported by a mudflow as evidenced by the nature of the sediments embedding the material. Although preferential fabric orientations in mudflows happen early in the transport, Bertran and Texier^38^ observed that they are more common in proximal parts, suggesting that the transport of the Kalinga material took place over a short distance. Furthermore, the absence of any imbricated clasts suggests that, although the flow was viscous, it was not turbulent^18^. This mudflow was somehow related to a volcanic eruption in the area as evidenced by the presence of volcanic glass in the Unit F silty-clay^7^ as well as of several unbroken wind transported volcanic quartz. The substrate on which the butchered rhino carcass rested was set in movement after this volcanic eruption, possibly because the vegetation was destroyed, or because of the heavy rain consecutive of such catastrophic events, or because of the two. This soil in movement transported the rhino bones to the main palaeochannel bed where the excavation focused on. This mudflow partly eroded the underlying sandy fluvial Unit A on its way, explaining the mix of purely aeolian and water transported quartzes in the Unit F. This mudflow not only transported the rhino but also covered it shortly after the butchery took place considering the good preservation of the skeleton.

In mudflows, fabrics are variable but tend for the majority of them to take a preferential orientation within a short distance and a small period of time^59,62^. Lindsay^59^ adds that planar fabrics in mudflows are the result of the instant at which the flow comes to a halt. Bimodal fabric distribution of orientations have also been observed for fluvial assemblages^21^. Elongate bones tend to orientate parallel to the current whenever the water volume exceeds the one of the bones, and parallel whenever this volume is less, such as in the case of water runoff^10,63^. In Kalinga, the elongate bones have been found to be statistically parallel to the southernmost portion of the main palaeochannel, and perpendicular to slope runoff. Runoff waters can result in planar fabrics but in these cases, the elements show no preferential orientation^33^ which is not the case in Kalinga as pointed out by the statistical tests rejecting isotropy. Furthermore, from the cluster analysis, it clearly appears that the distribution of the artefacts is not driven by the water catchment areas but mostly by the depth of the artefacts. If the water of the palaeochannels actively reworked the bones, then the clusters should reflect the catchment areas of the two independent streams. We suspect that the streams were passive in the deposition. The flow accumulation lines are logically the deepest parts of the palaeostreams bedding and this seems to be the reason why the objects accumulated there. The mudflow would have therefore naturally followed the stream beds filling the deepest parts of the topography first (Fig. 5a). This would explain why the artefacts within cluster 2 are arranged along the main streams direction.

The small fragments of isolated faunal remains had their own post-mortem history which differs from the rhino: fragments of turtle plastrons, a cervid radius and molars, two lamellae of a stegodon molar and monitor lizard femur and fibula. Their general colouration and the absence of black crusting for most of these fossils distinguish them from the rhino bones general aspect. Yet, this assemblage of isolated faunal remains cannot be regarded as one, although several accumulated in the same topographic feature of the excavation. The rodent marks observed on the *Varanus salvator* femur are evidence that this bone at least was, unlike the rhino, not buried immediately after the death of the animal. The slightly water abraded aspect of the *V. salvator* fibula witness yet another history for this bone. The two *V. salvator* remains most likely did not belong to the same individual. The turtle plastron in counterpart clearly belongs to one individual and their spread over the archaeological surface resulted into a different patterning with the rhino as they seem to follow the runoff along the topography slopes (Fig. 6a). Similarly, the grouping of the isolated cervid upper molars suggest, unlike the turtle, that they did not move much from their original position (Fig. 6a).

Because all the rhino bones, whatever their type is, have been recovered within a small catchment area and with a main orientation, experiments on transportability of the bones are of little use in Kalinga^53^. Larger bones tend to be deposited first although this is not a simple rule^64^. Attached ribs for instance have been observed to move further and faster^65^. In Kalinga, the attached ribs were found altogether in the same small area with the elongate bones suggesting similar speed and floatability.

The mud should have been viscous and the flow slow to transport such large and small bones without scattering them over a large area, and the distance for the transportation should have been short for the same reasons. The density pattern of small fragments of fauna which is located within the southern portion of the main palaeochannel and which does not overlap with the rhino bones density pattern is another support for the passive role of the palaeochannels. These small fragments of fauna further inform us on the limited competence of the main stream in which they have been recovered for most of them; the main flow was obviously incapable of transporting objects larger than 10 cm and heavier than 5 kg, which is the size and weight of the stegodon molar fragment, the largest and heaviest recovered piece from the main palaeochannel bed itself. Therefore, this flow could reasonably not be responsible for the transport of the large majority of the rhino bones. Additionally, the presence of rhino bones across the two catchment areas is another evidence. Overflowing of the palaeochannels could have resulted in such a dispersion of the remains, but then, one would not expect to find those remains within cluster 1 aligned along the palaeochannel beds. All these observations when considered altogether, supports our interpretation that the rhino carcass was transported by another agent than the streams, and most probably the mud covering the bones.

### Post-depositional evolution of the site and diagenesis

The presence of ashfalls evidenced by its weathering product, saponite, which was analyzed by XRD^7^, contributed, like fluvial and volcanic quartzes, to the acidification (lowered pH) of the sediments of Unit F after its deposition. This acidity combined with the anoxic conditions of the Unit F, resulted into the formation of hydromorphic features such as ferro-manganese concretions and crusts, but also probably into acid-forming minerals like the aluminium seen in the XRF analysis. These conditions, encrusting plus permanent water, certainly favored the preservation of the waterlogged wood fragment^7^ as well as the bones, although the acidity should have accelerated the deterioration of these latter in the absence of the crust.

Sporopollenin of pollen grains is sensitive to oxidation. Therefore, pollen grains are best preserved in redoxic environments, meaning waterlogged and anaerobic sediments such as clays, especially when acidic. Yet, although these conditions seem to have been present in Unit F, pollen grains were absent^7^. Similarly, volcanic ashes are considered to prevent pollen grains to decay^66,67^. Yet, in the famous Pompeii volcanic ashes, until the recent success of Weber and co-authors^68^ from very specific samples within human nasal cavities, fossil pollen grains extraction has been difficult for the longest time. Scholars^69^ considered that the heat of ashfall was not suitable for their preservation. It seems reasonable to hypothesize that the ashfall at Kalinga were still hot when they deposited over the soil on which the butchered rhino carcass lay. These hot ashes would not only have burnt the landscape, they would also have destroyed the pollen grains, explaining their absence within presumably favourable redox conditions. We observed no indications of burning at the surface of the bones. Yet, colouration by fire of bones largely depends on the temperature, and the duration of the burning^70^. Furthermore, the black crusting and orange-brown colouration of the rhino bones that developed at their surface once buried might have prevented the identification of any burnt surface.

### The relative age of the stone artefacts

Because the butchered rhino is certainly younger than any of the small faunal remains from the palaeochannel, one can wonder to what period of the site formation the stone artefacts are. Considering the burial of the rhino by the mudflow is the *terminus ante quem* of the Kalinga site’s Unit F and its constituent’s deposition, and that this event happened somehow immediately after the butchery of the carcass, the stone artefacts cannot be younger than the rhino.

Hence, three hypotheses can be drawn for the relative age of the stone artefacts at the Kalinga site:

1. The rhino carcass was transported by the mudflow into a catchment area within which diverse faunal fragments and stone artefacts had already been deposited.
2. The rhino carcass was transported along with the stone tools and the pebbles, as a secondary association due to the mudflow resulting in the mixing of the two assemblages, bones and stones.
3. The rhino carcass was transported along with the stone artefacts which were used to butcher the bones and their mixing is original. Both, the rhino and the artefacts would have then been transported altogether by the mudflow.

Hypothesis 1 can be rejected due to the different scattering pattern of the stone artefacts and the small isolated faunal fragments which is supported by statistical tests. While the latter were found in the bed of the main palaeochannel, the artefacts were spread all over the excavation area (Fig. 6e). The density pattern for the stone artefacts is the opposite to the rhino bones scattering. While the rhino main kernel is located within the main catchment area, the main kernel for the stone artefacts is located in the secondary catchment area. This can be explained by the size of the stone artefacts which are generally less than 50 mm. The smallest fragments of the rhino are, like the stone artefacts, located in the secondary catchment area. Similarly, hypothesis 2 can be rejected due to the different scattering pattern of the stone artefacts and the pebbles. This is true both, around the rhino elongated bones as evidenced by the LOESS regression curve, and for the whole excavated area as evidenced by the densities distribution and clustering pattern. Statistically, the stone artefacts are not correlated with the pebbles and they appear to be more correlated with the rhino scattering, although this correlation is not very strong, most likely for the above-mentioned reasons. Hypothesis 3 is far more difficult to be tested because of the numerous perturbations the Kalinga assemblage has undergone. The rejection of the two first hypotheses would lead us to consider hypothesis 3 as the most probable one. Although transportation and reorientation clearly happened to the material, it seems that the general relational properties between the rhino bones and the stone artefacts, recovered for a few of them inside the carcass and for most of them at the periphery of the carcass, are somewhat preserved. These relational properties are supported by statistical tests (Table 2 and Supplementary Figure 1), hence providing some space for interpreting the behaviour of the Kalinga butchers. The small stone artefacts would have been transported and were deposited along with the small rhino fragments undergoing a similar process.

The quantity of the cultural material lost during the Kalinga site formation is unknown. The absence of any refitting within the stone artefacts assemblage can be explained either by the loss of several elements during the short transport by the mudflow, or by the absence of any tool manufacture activities near the rhino carcass. A small number of stone artefacts are a common pattern of very large game butchery sites^71^.

Every single Pleistocene archaeological site is allochtonous when compared to the famous site of Pompeii^24^. The degree of post-depositional disturbance nevertheless differs from one site to another. The presence of the most fragile light bones along with the largest and heaviest bones suggests little transport of disassembled carcass parts from afar. Re-fitting of bone fragments within a small circumscribed area of the site is another evidence, as well as the similar surface pattern of all bones with the presence of butchery marks on a few bones. In this sense, the site of Kalinga can be regarded as an autochtonous butchery site with respect to the depositional place, following Behrensmeyer’s definition^9^, of a very large game for which the butchery marks have been recorded as well as the potential stone tools used for butchery. Yet, the small but clear depositional and post-depositional modification of the space surrounding the rhino prohibits us to have a direct access to the social organization of these early Middle Pleistocene Asian hominins from the Kalinga site.

## Supplementary information

Supplementary information
