# Supplementary material for: Taphonomy and chronosequence of the 709 ka Kalinga site formation (Luzon Island, Philippines)
Source: Sci Rep. 2020 Jul 6;10:11081. doi: 10.1038/s41598-020-68066-3 (PMC7338544; doi:10.1038/s41598-020-68066-3)
Supplement: Supplementary file 1 — Supplementary information [file 41598_2020_68066_MOESM1_ESM.docx]

**SUPPLEMENTARY INFORMATION**

**Taphonomy and Chronosequence of the 709 ka Kalinga Site Formation (Luzon Island, Philippines)**

T. Ingicco^1^, M.C. Reyes^2^, J. de Vos^3^, M. Belarmino^2^, P.C.H. Albers^3^, I. Lipardo^4^, X. Gallet^1^, N. Amano^5^, G. van den Bergh^6^, A.D. Cosalan^4^, A. Bautista^2^

*^1^ Département Homme et Environnement, UMR 7194, Muséum national d’Histoire naturelle, Sorbonne Université, Musée de l’Homme, 17 Place du Trocadéro, 75016 Paris, France*

*E-mail :* [*ingicco@mnhn.fr*](mailto:ingicco@mnhn.fr)

*^2^ National Museum of the Philippines, Padre Burgos St., Manila 1000, The Philippines*

*^3^ Naturalis Biodiversity Center, P.O. Box 9517, 2300 RA Leiden, The Netherlands*

*^4^ Archaeological Studies Program, Albert Hall, University of the Philippines, Diliman, Quezon City 1101, The Philippines*

*^5^ Max Planck Institute for the Science of Human History, Kahlaische Str. 10, Jena, 07745 Germany*

*^6^ Centre for Archaeological Science, School of Earth, Atmospheric and Life & Environmental Sciences, University of Wollongong, Wollongong, NSW 2522, Australia*


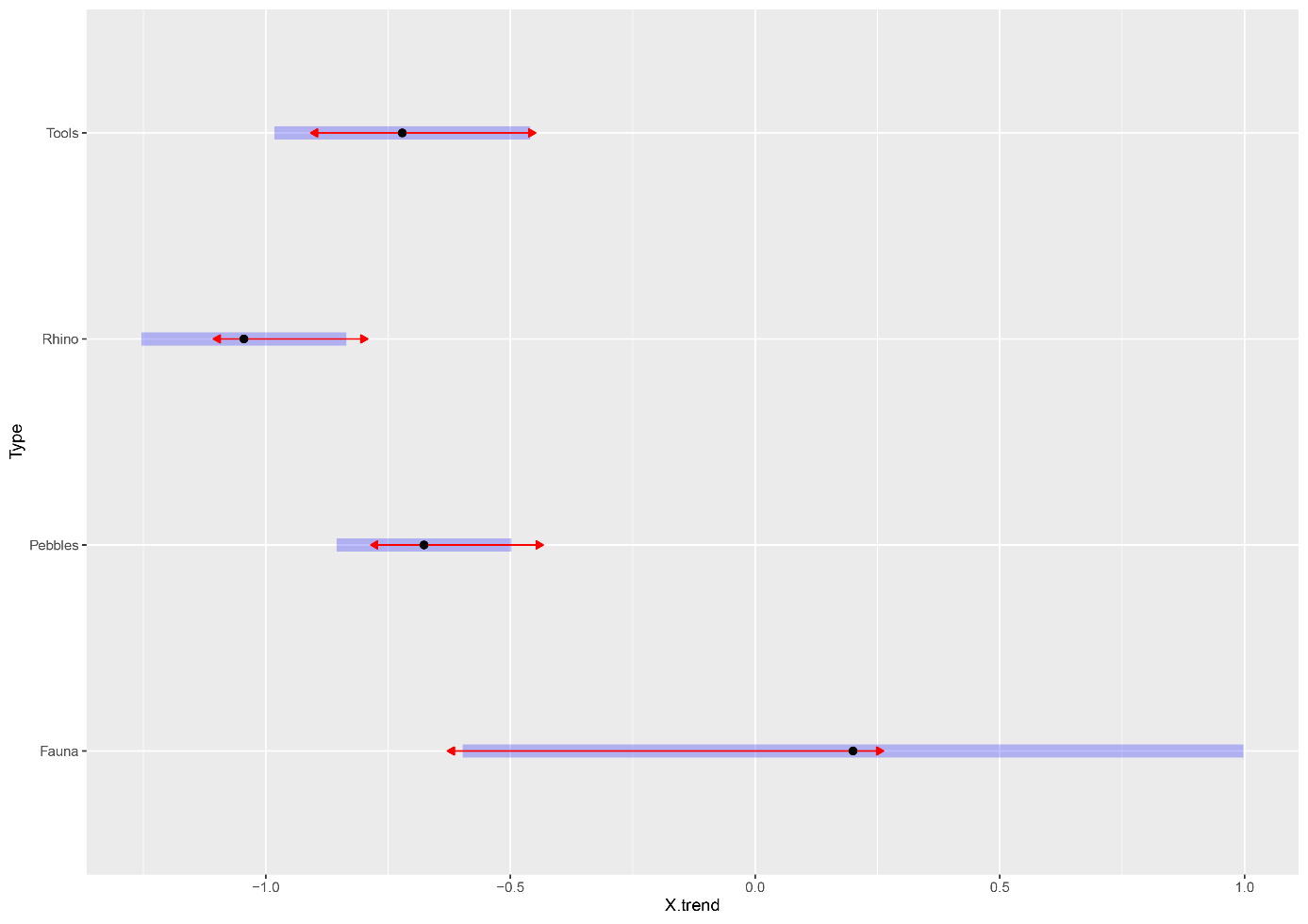


Supplementary figure 1 -- Estimated marginal means per category. The blue bars are confidence intervals for the Estimated Marginal Means, and the red arrows are for the comparisons among them. If an arrow from one mean overlaps an arrow from another group, the difference is not significant, based on Tukey test and the value of alpha (which defaults to 0.05). This is the case for the rhino density and the stone artefacts density.


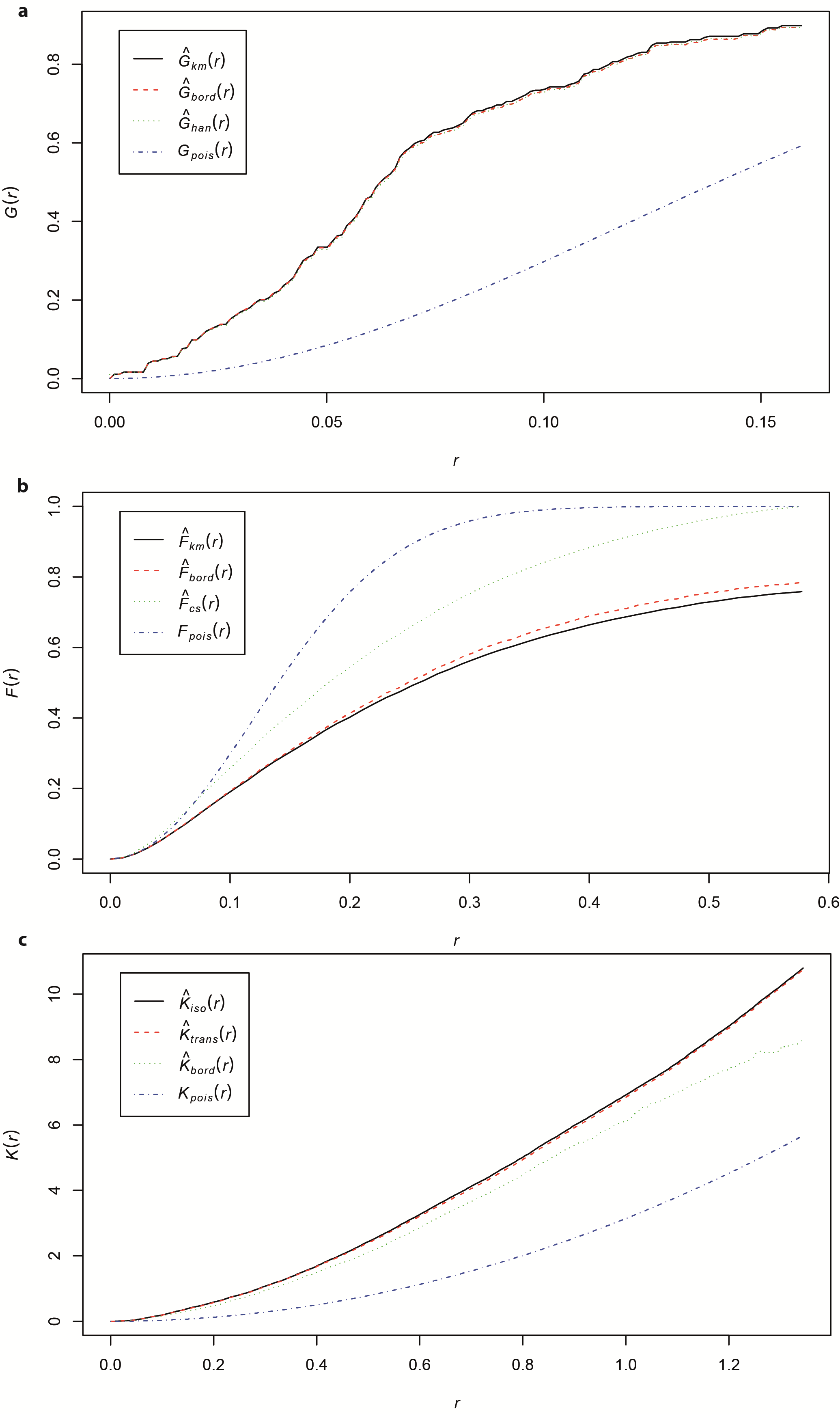


Supplementary figure 2 – G-function (a), F-function (b) and K-function (c) comparing the Kalinga assemblage to a theoretical one within the Poisson distribution and the nearest neighbor methods, validating the existence of clusters in the Kalinga assemblage.


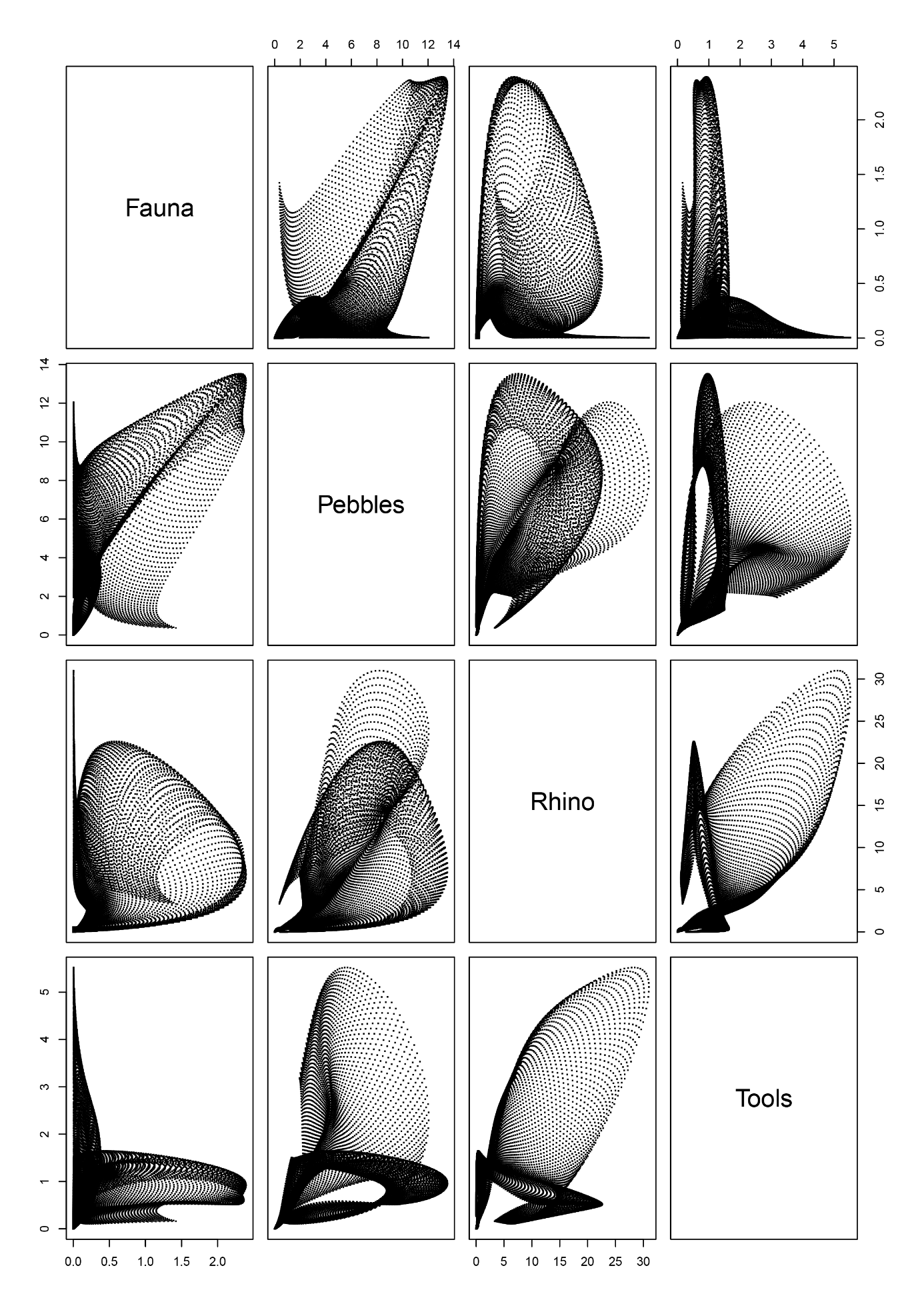


Supplementary figure 3 – Smoothed kernel intensity estimate for each category taken two by two.

Supplementary table 1 – Statistics for the fabric analysis
